# Pharmacological evaluation of Bioactive Principle of *Turnera aphrodisiaca*

**DOI:** 10.4103/0250-474X.49095

**Published:** 2008

**Authors:** S. Kumar, R. Madaan, A. Sharma

**Affiliations:** S. D. College of Pharmacy, Barnala-148 101, Punjab, India; 1 University Institute of Pharmaceutical Sciences, Panjab University, Chandigarh-160 014, India

**Keywords:** Anxiolytic, aphrodisiac, apigenin, *Turnera aphrodisiaca*, Turneraceae

## Abstract

In the present investigation, pharmacological evaluation of apigenin, a bioactive principle of *Turnera aphrodisiaca* Ward (Turneraceae) was carried out. Apigenin was evaluated for antianxiety activity at a dose of 2 mg/kg using well established models of anxiety, the hole board test, light/dark test and mirrored chamber test. Apigenin significantly increased head dipping in hole board test. Further, apigenin increased latency to leave light zone and the time spent in light compartment of light/dark model of anxiety. Apigenin also decreased the latency time to enter the mirrored chamber, and increased the total time spent/number of entries in the mirrored chamber with respect to control. All these observations confirmed the anxiolytic activity of apigenin. At a higher dose (about 12 fold the anxiolytic dose), apigenin showed mild sedative activity in actophotometer as it decreased activity scores. It (2, 5 or 10 mg/kg) was found to be devoid of anticonvulsant, antidepressant and antistress activity in MES-induced convulsion test, despair swim test and cold swimming endurance test, respectively. In tail immersion test for six hours, apigenin exhibited excellent dose dependent analgesic activity, which was comparable to that of morphine sulphate (5 mg/kg). Maximum activity was observed 30 min after the administration of 10 mg/kg dose of apigenin.

*Turnera aphrodisiaca* Ward (synonym *T. diffusa* Willd., family Turneraceae) is commonly known as damiana. The leaves of *T. aphrodisiaca* have been used traditionally as a stimulant, aphrodisiac, tonic, diuretic, nerve tonic, laxative and in kidney, menstrual and pregnancy disorders^1^,^2^. The British Herbal Pharmacopoeia^3^ lists specific indications for damiana as anxiety neurosis associated with impotency, and includes other indications such as depression, nervous dyspepsia, atonic constipation and coital inadequacy. Damiana has achieved some repute in the treatment of sexual impotence where it is used in conjunction with strychnine, phosphorus or some other stimulants in homoeopathic formulations^4^. The leaf infusion of damiana has been used as a traditional remedy in the diseases related to the gastrointestinal and respiratory system^5^, reproductive organs^6^, and for the treatment of gonorrhoea in Latin American societies^7^. Mother tincture (85% ethanol extract) of damiana is an important homoeopathic medicine for the treatment of sexual debility, and nervous prostration^8^.

Phytochemical reports on *T. aphrodisiaca* indicate that the plant contains tetraphyllin B (cyanoglycoside)^9^, gonzalitosin I (flavonoid)^10^, arbutin (phenolic glycoside)^11^, damianin^12^, tricosan-2-one, hexacosanol (hydrocarbons)^13^; a volatile oil containing α-pinene, β-pinene, p-cymene and 1,8-cineole^11^; and β-sitosterol (phytosterol)^10^.

A survey of literature on *T. aphrodisiaca* revealed only three pharmacological reports on the plant. Aqueous extract of *T. aphrodisiaca* whole plant has been reported to exhibit significant hypoglycaemic activity in alloxan-diabetic male mice^14^. Aguilara *et al.*^15^ have reported that decoction of *T. aphrodisiaca* leaves possesses significant hypoglycaemic activity in rabbits upon oral administration. Aqueous extract of the plant was reported to exhibit aphrodisiac activity in sexually sluggish male rats at a dose of 1 ml/kg^16^.

Recently we have reported that amongst various extracts viz., petroleum ether, chloroform, methanol and water of *T. aphrodisiaca* aerial parts, only methanol extract (25 mg/kg, p.o.) exhibited significant anti-anxiety activity on elevated plus maze apparatus^17^. An anxiolytic flavonoid, apigenin has been isolated from methanol extract of *T. aphrodisiaca* aerial parts using bioactivity-guided fractionation^18^. Present investigation was undertaken with an objective to develop pharmacological (anti-anxiety, sedative, anticonvulsant, antidepressant, antistress and analgesic) profile of apigenin isolated from *T. aphrodisiaca*.

## MATERIALS AND METHODS

Aerial parts of *T. aphrodisiaca* were procured from a cultivated source – Rati Ram Nursery, Village Khurrampur, district Saharanpur (UP) in the month of August 2002, and dried in shade. Identity of the plant was confirmed through Botanical Survey of India, Howrah. A voucher specimen of the plant has been deposited in the Herbarium-cum-Museum of the University Institute of Pharmaceutical Sciences, Panjab University, Chandigarh.

Laca mice (either sex), bred at the Central Animal House, Panjab University, Chandigarh, were allowed a standard pellet diet (Ashirwad, Chandigarh) and water *ad libitum*. Groups of five mice (20-24 g) were used in all sets of experiments. The animals were fasted for 18 h before use. The approval from the Institutional Animal Ethical Committee of Panjab University, Chandigarh was taken before carrying out biological studies.

Distilled water+Tween 80 (5%) were used as vehicle for preparing the suspension of various test doses of apigenin. Doses of apigenin were prepared by suspending in the vehicle in such concentrations as to administer these to mice in a volume ranging 0.20-0.24 ml per oral route. Diazepam (Triko Pharmaceuticals, Rohtak, Haryana), phenytoin sodium injections (Epsolin injections®; Zydus Neurosciences, Ahmedabad), imipramine (Triko Pharmaceuticals, Rohtak, Haryana) and morphine sulphate (Pharma Chemical Lab, Solan) were used as standard drugs.

### Evaluation of antianxiety activity:

The hole-board apparatus comprised a grey perspex panel (40×40 cm, 2.2 cm thick) with 16 equidistant holes (3 cm diameter) in the floor^19^,^20^. Photocells below the surface of the holes provided the measure of the number of head dips. The board was positioned 15 cm above the table and was divided with black water-resistant marker into 9 squares of 10×10 cm. Thirty minutes after the administration of the test drug, each mouse was individually placed in the centre of the board (facing away from the observer). During 5-min test period, number of head dips was noted.

The light/dark apparatus consisted of a rectangular box (46×27×30 cm), divided into one small (18×27 cm) and one large (27×27 cm) area, with an opening door 97.5×7.5 cm) located in the centre of the partition at floor level^19^. The small compartment was painted in black, whereas the large compartment was painted in white and brightly illuminated with 60 W cold light source. Thirty min after the administration of the test drug, each mouse was individually placed in the centre of the light compartment (facing away from the door). During 5-min test period, latency of the first crossing from light to dark compartment, and time spent in light zone were noted.

The mirrored-chamber apparatus consisted of a mirrored cube (30 cm on a side) open on one side that was placed inside a square wooden box (40×40×30.5 cm)^21^,^22^. The mirrored cube was constructed of five pieces of mirrored glass. The mirrors used were mirrored on one surface only (back surface being painted dark brown). The three mirrored side panes, a top pane, and the floor pane faced the interior of the cube. The mirrored cube was placed in the centre of the wooden container to form a 5 cm corridor that completely surrounded the mirror chamber. A mirror was also placed on the container wall so that it faced the single open side of the mirrored chamber. The other three walls of the container were painted dark brown. Thirty min after the administration of the test drug or standard, the mice were placed individually in the chamber of mirrors at a fixed corner. During 5-min test period, the following parameters were noted: (a) latency to enter the chamber, i.e., the time spent in seconds for the first entry into the chamber of mirrors and (b) the time spent with each entry was calculated by dividing the total time spent with number of entries.

### Evaluation of sedative activity:

Spontaneous locomotor activity was measured using an actophotometer (24×22×10 cm) (Popular Traders, Ambala) with automatic counting of animal movements on the activity cage floor^23^. Mice were placed individually in activity cage for 5-min test period, 30 min after administration of test drugs. Locomotor activity was observed in terms of the activity scores.

### Evaluation of anticonvulsant activity:

An electroconvulsive shock was applied through ear-clip electrodes of electroconvulsometer (IMP Corporation, Ambala) to induce tonic hindlimb extension in mice^24^. The maximal electroshock stimulus used for mice was 50 mA for 0.2 s. Mice were exposed to current, 45 min after the treatments. Time spent by the mice in tonic extensor phase of convulsions was recorded.

### Evaluation of antidepressant activity:

Mice were forced to swim, after 1 h of administration of test substances, in a plexiglas cylinder (height 40 cm; dia 18 cm) containing water up to the level of 15 cm, and maintained at 25±2°^25^. Mice were allowed to swim for 6 min. During this test period, the total duration of immobility (floating in the water in a slightly hunched but upright position, its nose above the surface) was noted.

### Evaluation of antistress activity:

Mice were forced to swim, after 1 h of administration of test drugs, in a plexiglas cylinder (height 40 cm; dia 18 cm) containing water up to the level of 15 cm, and maintained at 10±2°^26^,^27^. Mice were allowed to swim for 6 min. During this test period, the total duration of immobility was noted.

### Evaluation of analgesic activity:

Groups of mice were subjected to noxious stimulus (radiant heat) by placing 5 cm of the tail in a 500 ml beaker containing 450 ml water maintained at 55±2° before and after treatment with test drugs^28^. The tail withdrawal from the heat (flicking response) was taken as end point. A cut off period 15 s was observed to prevent damage to the tail. Three basal reaction times for each mouse at a gap of 5 min were taken to confirm normal behaviour of the mice. The reaction time at 30 min, 1 h, 2 h, 4 h and 6 h were recorded after the treatment. The percentage maximum possible effect (% MPE) is calculated from the formula, % MPE= (actual time–basal time)/(cut off time–basal time)

### Statistical analysis:

The results have been expressed as mean±standard error of mean (SEM). The test doses were compared with control by analysis of variance (P<0.05) followed by Studentized Tukey's test^29^.

## RESULTS AND DISCUSSION

**Fig. 1:**
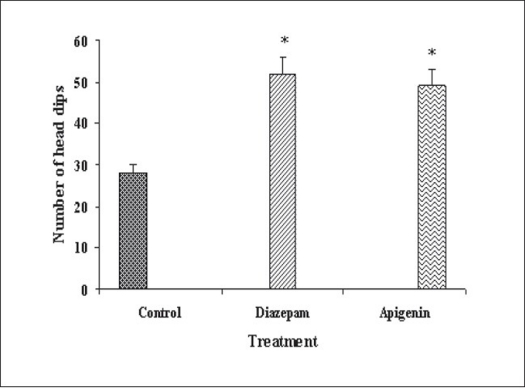
Antianxiety activity of apigenin using hole board test. The data is expressed as Mean±SEM of n=5 observations; *P<0.05 vs. control; ANOVA followed by Studentized Tukey's test. 
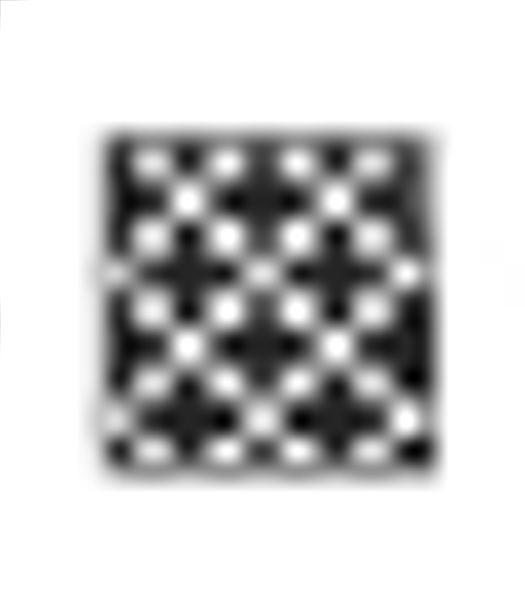
Vehicle; 
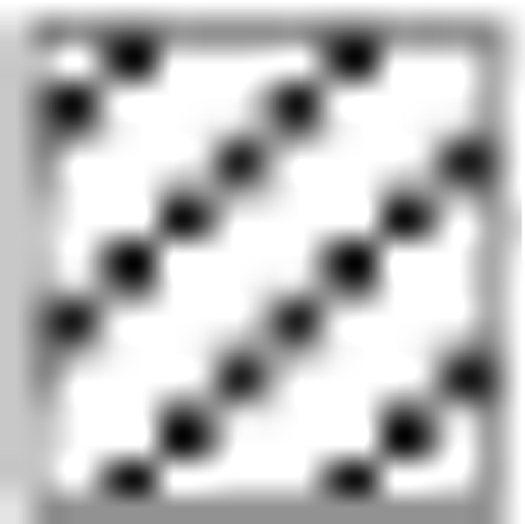
2 mg/kg; 
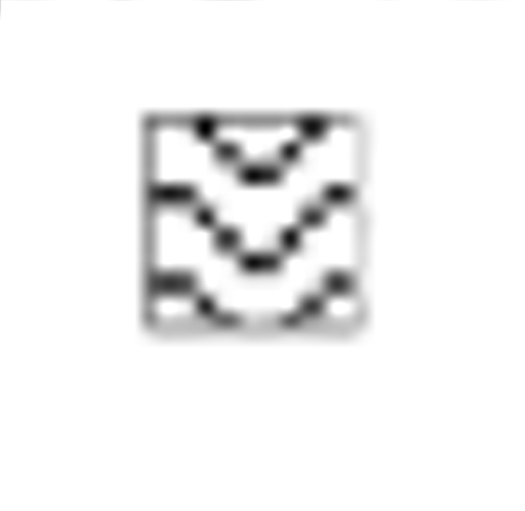
2 mg/kg

**Fig. 2:**
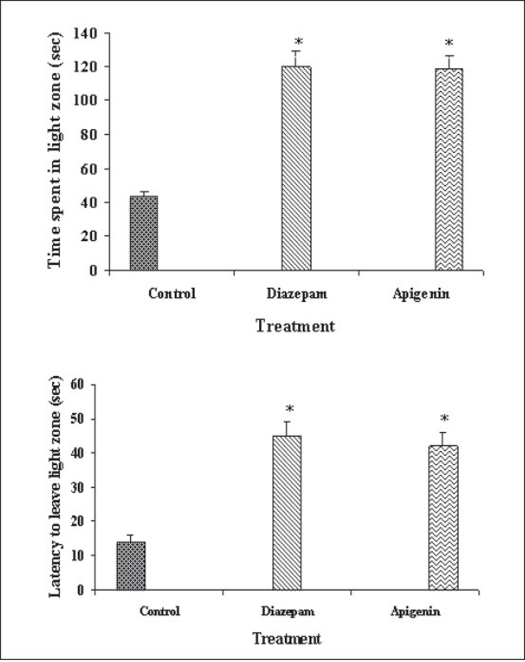
Antianxiety activity of apigenin using light/dark test. The data is expressed as Mean±SEM of n=5 observations; *P<0.05 vs. control; ANOVA followed by Studentized Tukey's test. 
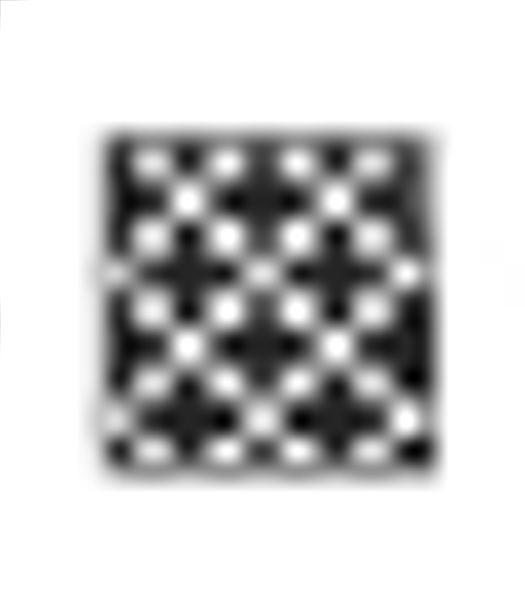
Vehicle; 
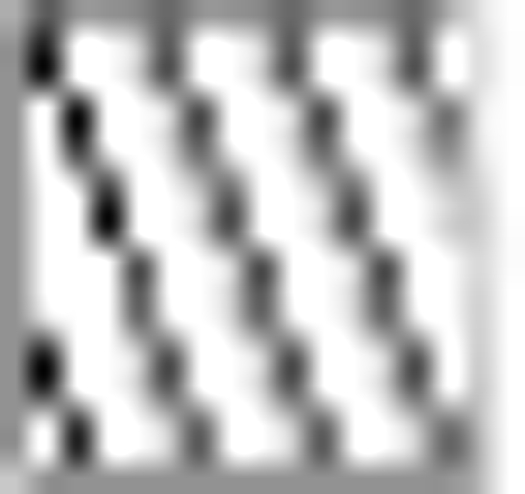
2 mg/kg; 
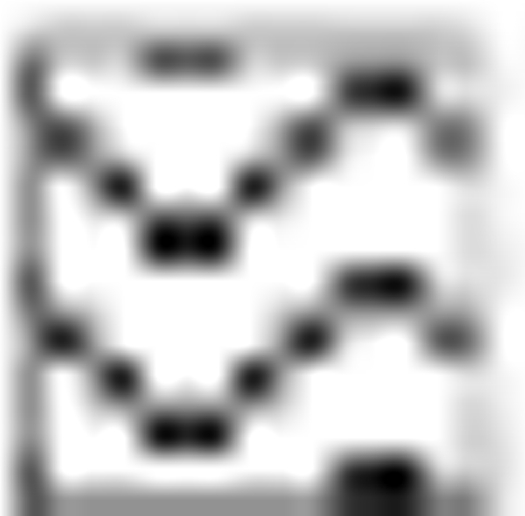
2 mg/kg

**Fig. 3:**
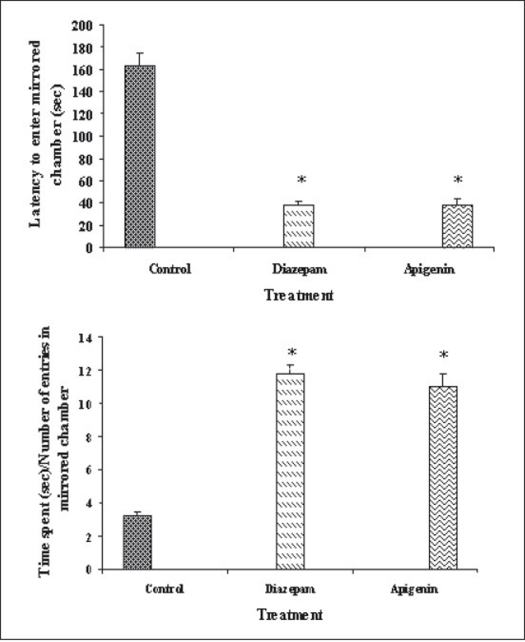
Antianxiety activity of apigenin using mirrored chamber test. The data is expressed as Mean±SEM of n=5 observations; *P<0.05 vs. control; ANOVA followed by Studentized Tukey's test. 
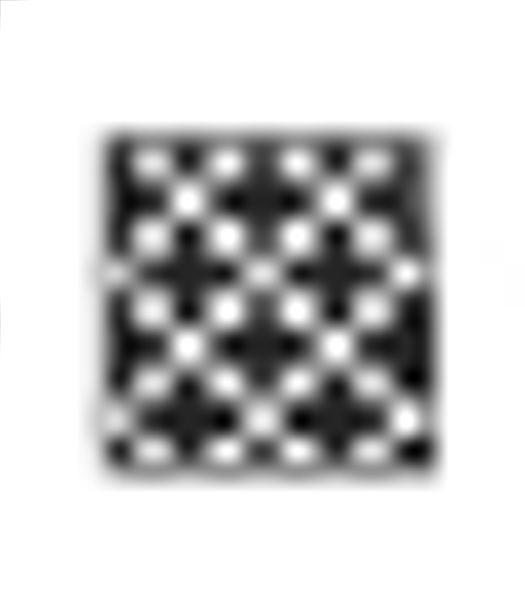
Vehicle; 
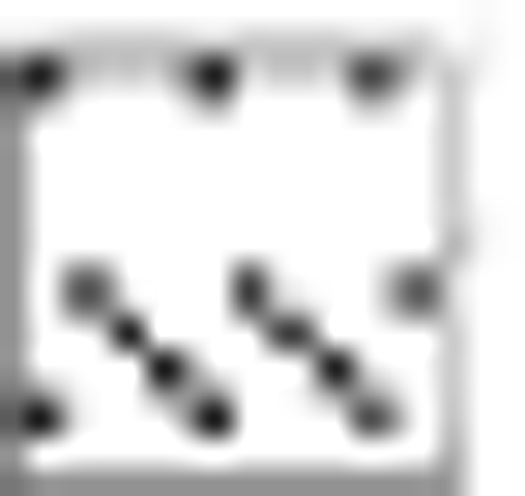
2 mg/kg; 
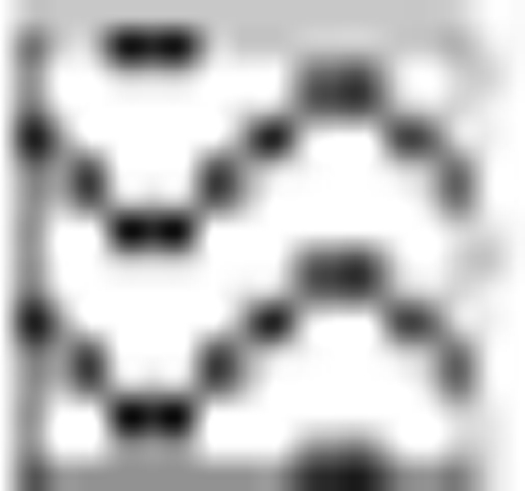
2 mg/kg

Our previous work based on bioactivity-directed fractionation of bioactive methanol extract of *T. aphrodisiaca* aerial parts afforded an anxiolytic flavone, apigenin which exhibited significant anti-anxiety activity at a dose of 2 mg/kg using elevated plus maze model of anxiety^18^. To substantiate data, apigenin was further evaluated for antianxiety activity in other well established models of anxiety, and also for various CNS-related bioactivities. Apigenin was evaluated for anti-anxiety activity at a dose of 2 mg/kg using well established models of anxiety, i.e., hole board test, light/dark test and mirrored chamber test. Results of hole board, light/dark and mirrored chamber tests have been shown in figs. 1–3, respectively. Apigenin significantly increased head dipping in hole board test. Further, apigenin increased latency to leave light zone and the time spent in light compartment of light/dark model of anxiety. Apigenin also decreased the latency time to enter the mirrored chamber, and increased the total time spent/number of entries in the mirrored chamber with respect to control. All these observations confirmed the anxiolytic activity of apigenin.

**Fig. 4:**
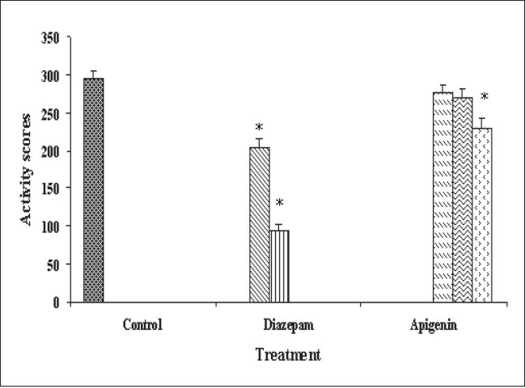
Sedative activity of apigenin using actophotometer. The data is expressed as Mean±SEM of n=5 observations; *P<0.05 vs. control; ANOVA followed by Studentized Tukey's test. 
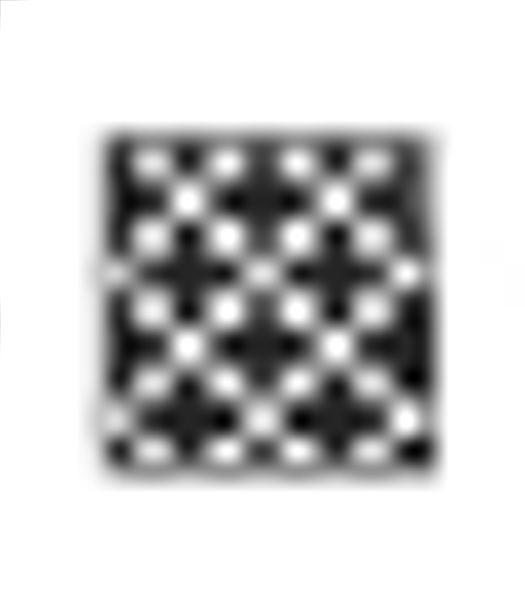
Vehicle; 
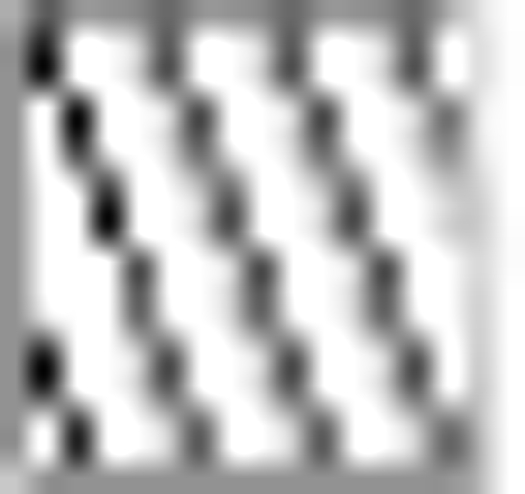
5 mg/kg; 
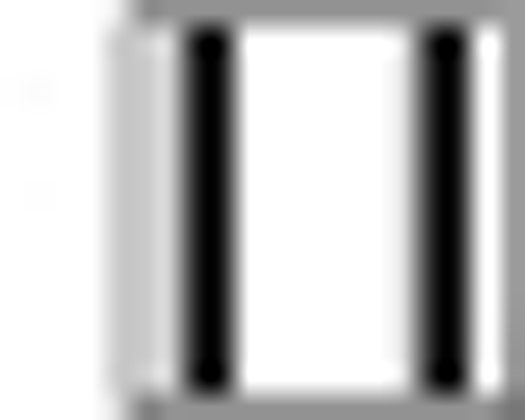
10 mg/kg; 
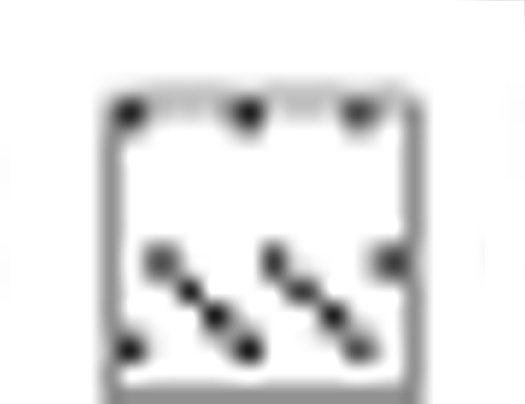
5 mg/kg; 
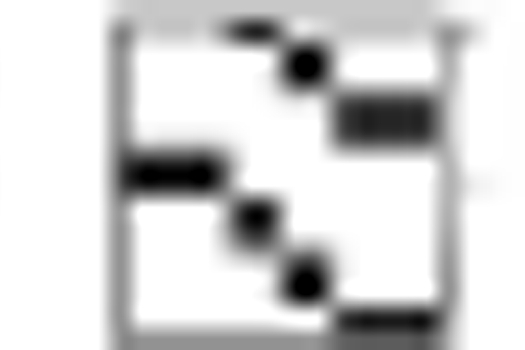
10 mg/kg; 
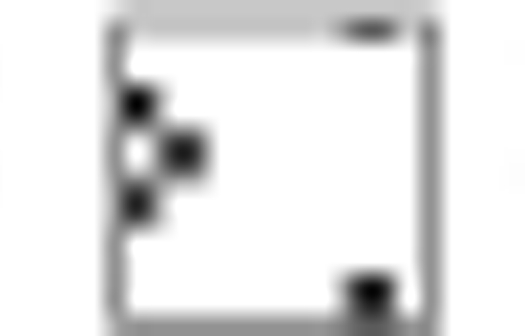
25 mg/kg

Apigenin was evaluated for sedative activity using actophotometer apparatus at doses of 5, 10 or 25 mg/kg, and results have been shown in fig. 4. At a higher dose 25 mg/kg (about 12 fold the anxiolytic dose), apigenin showed mild sedative activity in actophotometer as it decreased activity scores. The results were statistically significant to that of control group but not comparable to diazepam (10 mg/kg). Apigenin did not exhibit sedative activity at doses of 5 or 10 mg/kg.

Apigenin was evaluated for anticonvulsant activity using MES apparatus at doses of 5, 10 or 25 mg/kg, antidepressant activity using despair swim test at dose levels of 2, 5 or 10 mg/kg, and for antistress activity using cold swimming test at doses of 2, 5 or 10 mg/kg. It was found to be devoid of anticonvulsant, antidepressant and antistress activity at all tested doses.

**Fig. 5:**
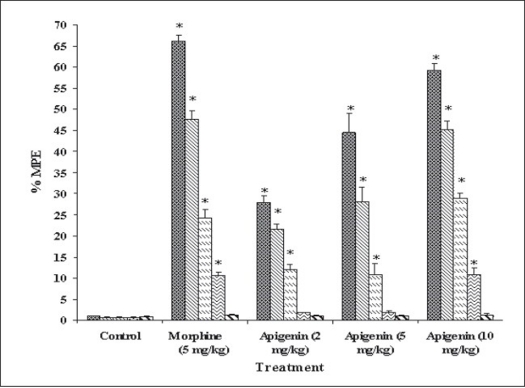
Analgesic activity of apigenin in tail immersion test The data is expressed as Mean±SEM of n=5 observations; *P<0.05 vs. control; ANOVA followed by Studentized Tukey's test. 
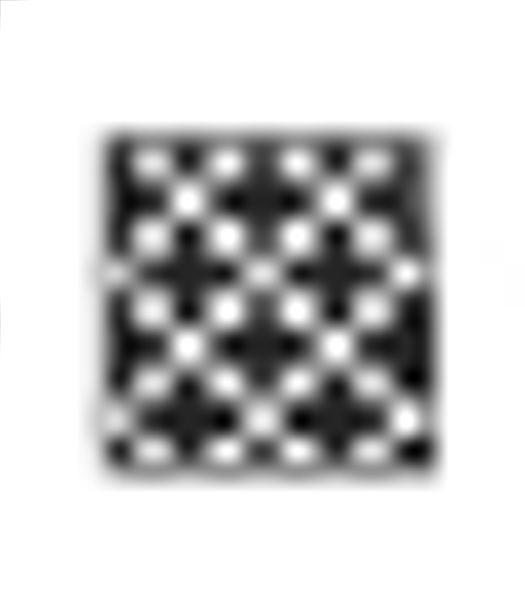
30 min; 
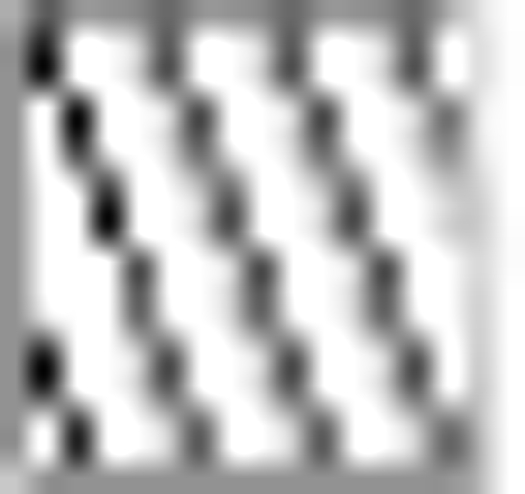
60 min; 
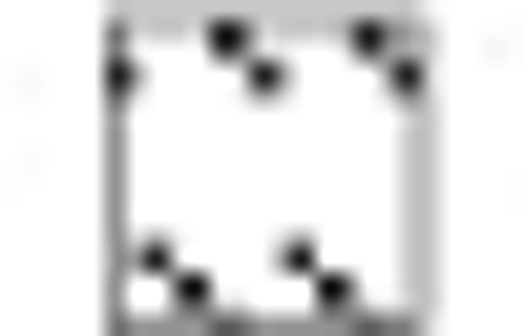
120 min; 
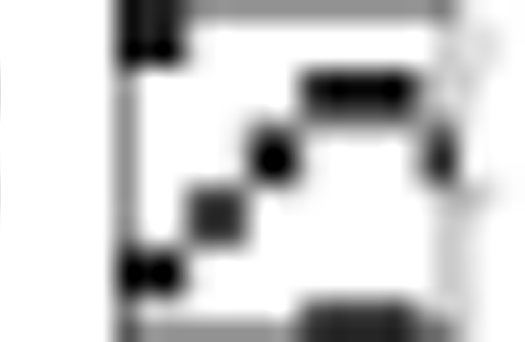
240 min; 
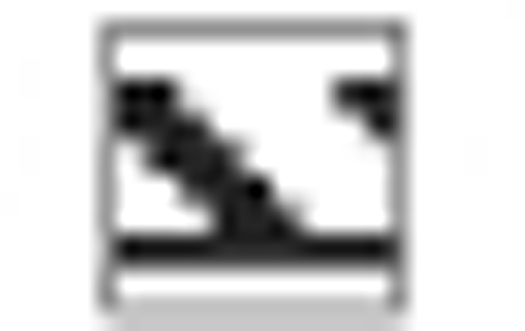
360 min

Apigenin was evaluated for analgesic activity using tail immersion test at doses of 2, 5 or 10 mg/kg, and results have been shown in fig. 5. In tail immersion test for 6 h, apigenin exhibited excellent analgesic activity, which was comparable to that of morphine sulphate (5 mg/kg). Apigenin (2, 5 or 10 mg/kg) showed dose-dependent analgesic activity. Maximum activity was observed 30 min after the administration of 10 mg/kg dose of apigenin. The activity remained significant up to the 4^th^ h of study in a manner similar to that shown by morphine sulphate. At 6^th^ h, analgesic activity decreased which might be due to the metabolism of apigenin.
